# The “Intermediate” CD14^++^CD16^+^ monocyte subset increases in severe peripheral artery disease in humans

**DOI:** 10.1038/srep39483

**Published:** 2016-12-19

**Authors:** Moritz Wildgruber, Teresa Aschenbrenner, Heiko Wendorff, Maria Czubba, Almut Glinzer, Bernhard Haller, Matthias Schiemann, Alexander Zimmermann, Hermann Berger, Hans-Henning Eckstein, Reinhard Meier, Walter A. Wohlgemuth, Peter Libby, Alma Zernecke

**Affiliations:** 1 Institut für diagnostische und interventionelle Radiologie, Klinikum rechts der Isar, Technische Universität München, Germany; 2 Institut für Klinische Radiologie, Universitätsklinikum Münster, Germany; 3 Klinik für vaskuläre und endovaskuläre Chirurgie, Klinikum rechts der Isar, Technische Universität München, Germany; 4 Institut für medizinische Statistik und Epidemiologie, Klinikum rechts der Isar, Technische Universität München, Germany; 5 Institut für medizinische Mikrobiologie, Immunologie und Hygiene, Technische Universität München, Germany; 6 Klinische Kooperationsgemeinschaft “Immunmonitoring”, Helmholtz Zentrum München (Neuherberg) und Technische Universität München, Germany; 7 Institut für Radiologie, Universitätsklinikum Ulm, Germany; 8 Institut für Röntgendiagnostik, Universitätsklinikum Regensburg, Germany; 9 Cardiovascular Division, Department of Medicine, Brigham and Women’s Hospital, Harvard Medical School, Boston, USA; 10 Institut für Klinische Biochemie und Pathobiochemie, Universitätsklinikum Würzburg, Germany

## Abstract

Monocytes are key players in atherosclerotic. Human monocytes display a considerable heterogeneity and at least three subsets can be distinguished. While the role of monocyte subset heterogeneity has already been well investigated in coronary artery disease (CAD), the knowledge about monocytes and their heterogeneity in peripheral artery occlusive disease (PAOD) still is limited. Therefore, we aimed to investigate monocyte subset heterogeneity in patients with PAOD. Peripheral blood was obtained from 143 patients suffering from PAOD (Rutherford stage I to VI) and three monocyte subsets were identified by flow cytometry: CD14^++^CD16^−^ classical monocytes, CD14^+^CD16^++^ non-classical monocytes and CD14^++^CD16^+^ intermediate monocytes. Additionally the expression of distinct surface markers (CD106, CD162 and myeloperoxidase MPO) was analyzed. Proportions of CD14^++^CD16^+^ intermediate monocyte levels were significantly increased in advanced stages of PAOD, while classical and non-classical monocytes displayed no such trend. Moreover, CD162 and MPO expression increased significantly in intermediate monocyte subsets in advanced disease stages. Likewise, increased CD162 and MPO expression was noted in CD14^++^CD16^−^ classical monocytes. These data suggest substantial dynamics in monocyte subset distributions and phenotypes in different stages of PAOD, which can either serve as biomarkers or as potential therapeutic targets to decrease the inflammatory burden in advanced stages of atherosclerosis.

Atherosclerosis, a chronic inflammatory disease of the arterial wall, remains the underlying cause of cardiovascular complications such as myocardial infarction, stroke, and peripheral artery occlusive disease (PAOD)^1^. Despite improvement in the interventional and pharmacological treatment of atherosclerosis, this disease remains a leading cause of death in developed countries. A deeper understanding of its cellular and molecular mechanisms could aid the development of tailored therapies for atherosclerosis and its complications. Local accumulation of leukocytes helps drive atherosclerotic lesion formation, and during the last decade monocytes have gained growing attention as key contributors to atherogenesis^2^. Beyond their role in initial lesion formation, monocytes also participate in the progression of atherosclerotic lesions, and the precipitation of thrombotic complications^3^.

Monocytes display considerable heterogeneity. Their subsets, defined by surface markers both in mice and humans, show distinct and divergent functions and play specialized roles in the formation and propagation of atherosclerotic lesions^2^,^4^. In humans, monocyte subsets differ in their expressions of the LPS receptor CD14 and the FcγIII receptor CD16. CD14^++^CD16^−^ monocytes, frequently described as classical monocytes, dominate in the peripheral circulation, compared to ‘non-classical’ CD14^+^CD16^++^. More recent work has identified an additional intermediate monocyte subset: CD14^++^CD16^+^ monocytes numerically represent the smallest monocyte population. This subset, however, has gained interest as it can secrete high amounts of TNF-α in response to LPS stimulation^5^. Cluster analysis has revealed that this intermediate subset closely relates to CD16^−^ monocytes and resembles proinflammatroy murine Ly6C^hi^/Gr-1^+^ rather than Ly6C^low^/Gr-1^−^ monocytes^4^,^6^,^7^.

Various clinical studies in patients with coronary artery disease (CAD) or myocardial infarction have evaluated monocyte subsets. Either CD14^++^CD16^+^ or CD14^++^CD16^−^ monocytes can independently predict future cardiovascular events and the outcome after myocardial infarction^8^,^9^,^10^,^11^.

In addition to coronary artery disease, PAOD remains a major clinical manifestation of atherosclerosis. PAOD causes debilitating intermittent claudication and limb ischemia, which can progress to gangrene and tissue necrosis, ultimately requiring amputation, particularly in diabetic populations. A recent study in Germany has revealed that treatment outcomes remain poor, particularly in patients with critical limb ischemia (CLI), resulting in high rates of amputations predominantly in patients with advanced disease as indicated by a higher Rutherford category. Additionally, a higher Rutherford scale associated with increased rates of myocardial infarction, stroke, and death, providing evidence that PAOD serves as marker of disease severity and predictor for other cardiovascular events beyond its typical peripheral clinical manifestations^12^. This disease causes not only limitations in mobility and impaired quality of life, but contributes to increased health care expenditures.

Despite previous investigations focused primarily on coronary atherosclerosis, the role of monocytes and their heterogeneity in PAOD remains only poorly understood. The current study prospectively assessed monocyte levels as well as monocyte subset distributions and phenotypes in patients with various degrees of atherosclerosis of the lower limbs, and tested correlations with the severity of PAOD, as assessed by the Rutherford score. This work aimed to gain mechanistic insight as well as potential novel biomarkers for progression of this understudied but prevalent form of atherosclerosis.

## Results

### Patient characteristics

Enrollment of 143 patients (94 males, 49 females; mean age 72 ± 10 years) with various degrees of PAOD occurred between October 2012 and January 2014. Table 1 shows patient characteristics and lesion distributions, differentiated according to the Rutherford classification. Some risk factors, including diabetes, tobacco use, concomitant malignancy, as well as statin medication (20 mg Atorvastastin daily) showed significant differences between the Rutherford stages. To adjust for bias induced by these confounders, all further analyses were adjusted to the distribution of those risk factors.

### Leukocyte and monocyte counts

**Figure 1:**
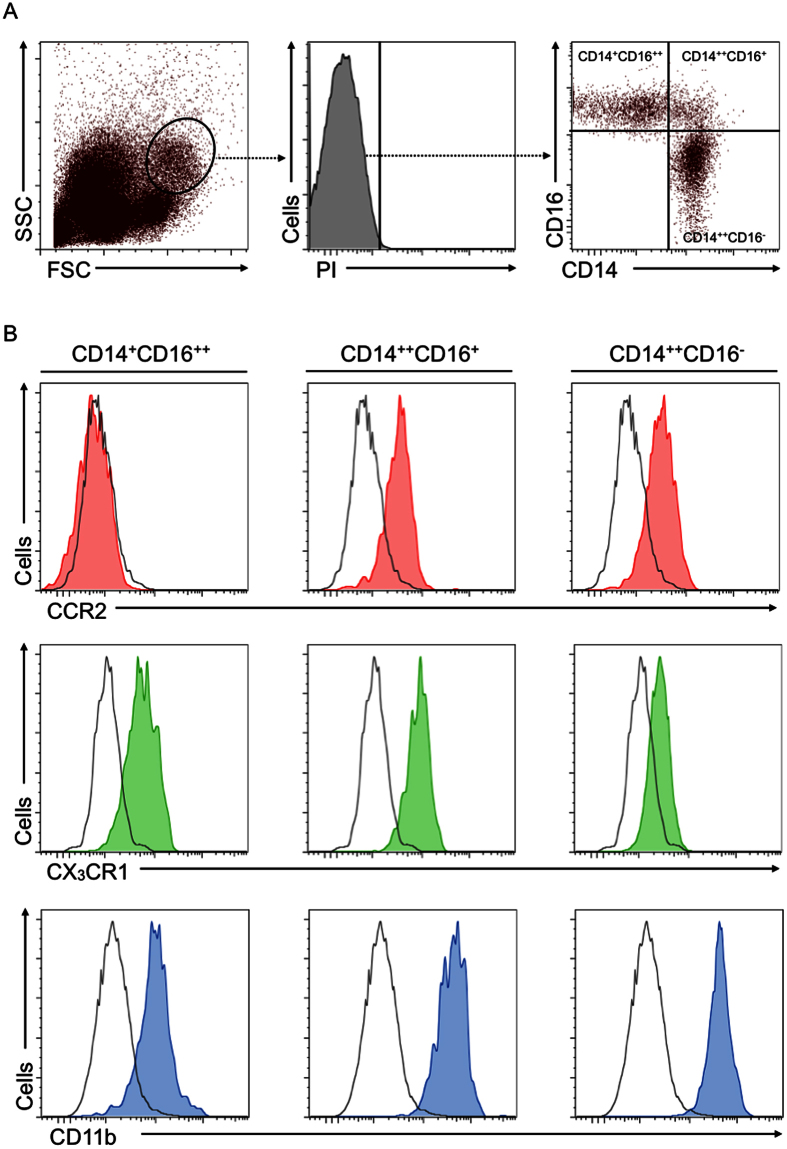
Characterization of monocyte subsets by flow cytometry. Panel A: Human monocytes were identified by flow cytometry by their appearance on the forward-sideward-scatter. Dead cells were excluded by propidium iodine staining. Three major monocyte subsets were identified by their expression of CD14 and CD16. Representative dot plots and histograms are shown. Panel B: Expression of CCR2, CX_3_CR1 and CD11b were assessed in each of the monocyte subsets and expression levels are depicted in representative histograms; black curve indicate isotype control staining.

Laboratory testing obtained leukocyte counts. Flow cytometry applying established gating strategies identified monocytes as well as monocyte subset populations. Flow cytometric analyses identified monocytes by their profile on forward versus side scatter dot plots, after elimination of dead cells determined by propidium iodide staining. These procedures discriminated the three major monocyte subsets by their expression of CD14 and CD16. “Non-classical” CD14^+^CD16^++^ monocytes‚ “intermediate” CD14^++^CD16^+^ double-positive monocytes, and “classical” CD14^++^CD16^−^ monocytes were further characterized with respect to their expression of CCR2, CX_3_CR1, and CD11b (Fig. 1). As expected, CD14^++^CD16^−^ monocytes showed high expression of CCR2 but low expression of CX_3_CR1. CD14^++^CD16^+^ monocytes remained highly positive for both of these chemokine receptors, and CD14^+^CD16^++^ monocytes showed low CCR2 but high CX_3_CR1 expression. All subsets showed strong reactivity for CD11b.

**Figure 2:**
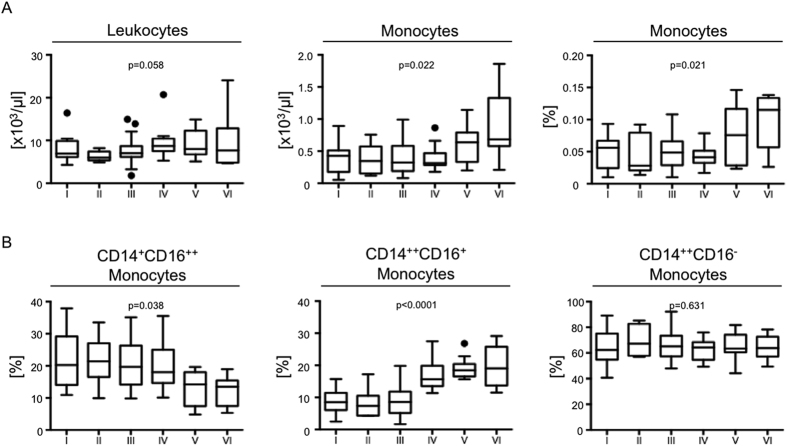
Leukocytes, monocytes and monocyte subsets in PAOD patients. Total leukocyte and monocyte counts, as well as monocyte frequencies among leukocytes (Panel A) as well as monocyte subset distributions among total monocytes (**B**) were assessed in n = 143 patients with various degrees of PAOD, as represented by different Rutherford stages (I–VI). Relationships between Rutherford stage and the different cell measures were assessed using Pearson’s correlation. A significant correlation is indicated by a p-value < 0.05. Data are presented as box plots, outliers are shown as single dots. Sample sizes are: Rutherford stage I: n = 24, II: n = 8, III: n = 74, IV: n = 16, V: n = 13, VI: n = 8.

Analysis of leukocyte as well as monocyte levels in PAOD patients tested associations with different Rutherford stages (Fig. 2A). Total peripheral blood leukocyte counts showed only a moderate, but only borderline significant increase with higher Rutherford stages (r = 0.208, p = 0.058). In contrast, absolute monocyte counts expanded in patients with more advanced stages of the disease and associated with disease severity (r = 0.249, p = 0.022). Similarly, frequencies of total monocytes among leukocytes increased in patients with advanced PAOD (r = 0.246, p = 0.021).

### Monocyte Subset Distributions

Further analyses determined the distribution of different monocyte subsets in these patients (Fig. 2B). “Classical” CD14^++^CD16^−^ monocytes showed no relevant alterations between different stages of PAOD (r = −0.053, p = 0.631). The proportion of “non-classical” CD14^+^CD16^++^ monocytes, however, showed a decrease in patients with advanced PAOD (r = −0.227, p = 0.038), and remained particularly low in patients with Rutherford stage V and VI disease. In contrast, frequencies of “intermediate” CD14^++^CD16^+^ monocytes increased with more advanced disease stages (r = 0.501, p < 0.0001), with a major increase in patients with stage IV disease (ischemic rest pain) compared to patients with Rutherford stage III (intermittent claudication). To differentiate between the potential of monocyte subsets to serve as a biomarker for PAOD progression specifically or more generally a disease progression of atherosclerosis, we compared all n = 143 patients suffering from PAOD to patients suffering from PAOD only (n = 82), and excluding those with coronary and carotid artery disease (n = 61). Due to the decreased sample size we summarized PAOD stages with a similar trend, respectively Rutherford stage I to III and stage IV to VI. The results showed that subset distributions with respect to the “intermediate” CD14^++^CD16^+^ monocytes as well as the “classical” CD14^++^CD16^−^ monocytes were similar between patients suffering from PAOD only, compared to patients suffering from PAOD and additionally from coronary and/or carotid artery disease. Only the decrease in “non-classical” CD14^+^CD16^++^ monocytes in advanced disease stages was more profound in patients suffering from generalized atherosclerosis compared to patients suffering from PAOD only (Supplementary Figure 1).

### Phenotypic characterization of monocyte subsets

Further assessment of the expression of characteristic surface structures involved comparing their mean fluorescence intensity (MFI) on different subsets by flow cytometry. The expression of CCR2, CX_3_CR1, and CD11b did not change significantly between different clinical stages of PAOD (data not shown), indicating no relevant changes in phenotype with respect to these markers.

**Figure 3:**
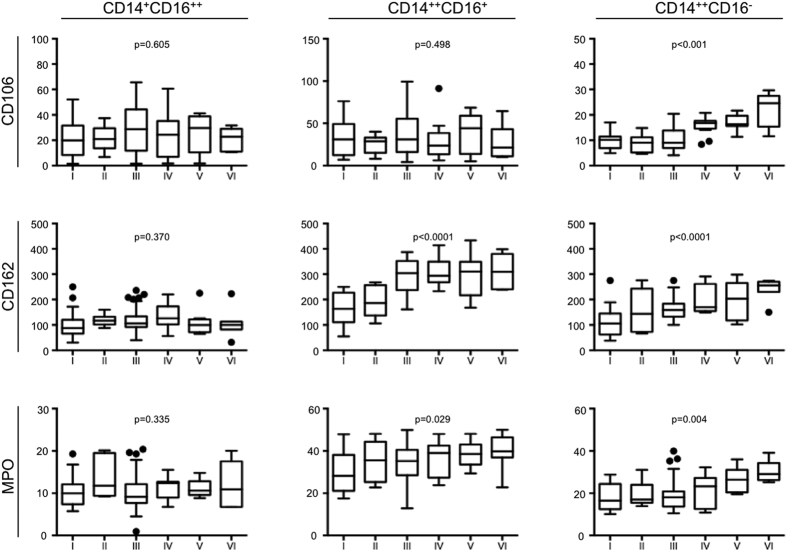
CD106, CD162 and MPO expression in monocyte subsets. Surface expression of CD106/VCAM-1 (Panel A) and CD162/PSGL-1 (**B**) and MPO content (**C**) were assessed by flow cytometry in each of the three monocyte subsets. Significant increase/decrease of biomarker expression/content are indicated by a p-value < 0.05. Data are presented as box plots, outliers are shown as single dots. Sample sizes are: Rutherford stage I: n = 24, II: n = 8, III: n = 74, IV: n = 16, V: n = 13, VI: n = 8.

Yet, patients with advanced stages of PAOD suffering from ischemic rest pain or gangrene/tissue loss had significantly increased expression of CD106 on “classical” CD14^++^CD16^−^ monocytes (r = 0.559, p < 0.001.) CD106 expression on “non-classical” CD14^+^CD16^++^ as well as “intermediate” CD14^++^CD16^+^ monocytes did not change significantly in PAOD (Fig. 3A)”.

CD162 (P-selectin glycoprotein ligand 1) constitutes an important adhesive molecule on monocytes. CD162 expression increased significantly in both “intermediate” CD14^++^CD16^+^ (r = 0.479, p < 0.001) as well as “classical” CD14^++^CD16^−^ monocytes (r = 0.584, p < 0.001), but not in “non-classical” CD14^+^CD16^++^ monocytes (Fig. 3B).

Similarly, myeloperoxidase (MPO) content, identified by intracellular staining, increased significantly in “intermediate” CD14^++^CD16^+^ (r = 0.238, p = 0.029) as well as “classical” CD14^++^CD16^−^ monocytes (r = 0.310, p = 0.004), while “non-classical” CD14^+^CD16^++^ monocytes showed no changes in patients with increasing PAOD disease severity (Fig. 3C).

## Discussion

Despite advances in treatment strategies, current clinical outcomes remain poor in patients with PAOD. Besides the disabling character of PAOD itself, the disease can also associate with life-threatening cardiovascular events, such as stroke or myocardial infarction^12^. While much research investigating atherosclerosis focuses on the coronary or carotid arteries, fewer studies have addressed the biology of PAOD. Compared to the coronary or carotid artery, characterization of the lower limb vasculature involves different blood flow patterns, the potential of extensive collateral growth after occlusion of a major artery, and in general a much larger vessel length susceptible to atherosclerosis. Various investigations have sought indicators for disease progression, especially from the stage of intermittent claudication to critical limb ischemia^13^,^14^,^15^, and the identification of markers of inflammation that associate significantly with advanced stages of PAOD^14^,^16^. Monocytes contribute critically to the initiation, progression, and thrombotic complications of atherosclerosis, and may serve as predictors of cardiovascular events in humans^1^,^4^. Yet, scant data exists regarding their potential contribution to PAOD. We have therefore investigated monocyte subset distributions and phenotype in patients with PAOD.

The current study shows that patients with advanced stages of PAOD, reflected by a higher Rutherford score, exhibit increased numbers of circulating monocytes. In particular, monocyte counts increased in patients with gangrene and tissue loss. These results may reflect in part a response to tissue injury. More interestingly, our results showed changes in monocyte subset distributions at earlier stages of disease, before the onset of ischemic injury to tissues. A significantly increased percentage of intermediate CD14^++^CD16^+^ monocytes occurred in patients with ischemic rest pain compared to intermittent claudication, correlating with disease progression. At the same time the fraction of non-classical CD14^+^CD16^++^ monocytes decreased in patients with Rutherford stage V and VI PAOD. Non-classical CD14^++^CD16^−^ monocytes did not differ between moderate and advanced stages of PAOD. The variations in monocyte subset distributions may reflect differences in monocyte subset production or consumption.

In patients with suspected coronary artery disease as well as in patients with chronic kidney disease, intermediate CD14^++^C16^++^ monocytes independently predicted future cardiovascular events in large patient cohorts^9^,^10^ and correlated with markers of inflammation and fibronolysis^17^. In addition, previous data suggests that the intermediate subset secretes increased amounts of TNF-α in patients with CAD and therefore can exert proinflammatory functions^5^. Previous reports indicate that classical CD14^++^CD16^−^ monocytes, however, can predict cardiovascular events in a randomly selected subject population within the Malmö Diet and Cancer Study^8^ and associate with subclinical systemic atherosclerosis and increased carotid intima-media thickness^18^. In addition, patient risk factors may affect monocyte subset populations. Non-classical CD14^+^CD16^++^ as well as intermediate CD14^++^CD16^+^ monocytes both increase in obese patients, whereas the CD14^+^CD16^++^ subset remains significantly enriched in obese diabetic patients^19^. These studies suggest that the disease stage and certain risk factors can differentially affect specific monocyte subsets. The use of these subsets as biomarkers therefore merits cautious evaluation with the course of the disease and individual risk factors taken into account.

With the work on monocyte subsets in cardiovascular disease in mind, the intermediate CD14^++^CD16^+^ monocytes can doubtless be seen as a subset with a proinflammatory functions. The increase in CD14^++^CD16^+^ monocytes in patients with more advanced stages of PAOD, observed in the present study, may thus indicate PAOD progression. Supported by findings that CD14^++^CD16^+^ monocytes predict cardiovascular events in patients with increased risk for CAD^9^,^10^, these findings agree with the propensity of patients with PAOD to have events in the distributions of other arterial beds^12^. The decrease in CD14^+^CD16^++^ non-classical monocytes in the patients with advanced stages of PAOD displaying ischemic gangrene and tissue loss may indicate an impaired healing response, given that data has shown that this subset rather plays important roles in tissue homeostasis and repair^20^. Replenishing these potentially reparative CD14^+^CD16^++^ monocytes might provide a novel therapeutic option. Evidence that mononuclear cell transfer can mediate exercise-induced collateral artery growth in mice illustrates monocyte transfer as an amenable therapeutic approach^21^.

An assessment of the expression of the characteristic phenotypic markers CD11b, CX_3_CR1, and CCR2 helped to characterize further the monocyte subset response in patients with different stages of PAOD. We found no changes of these markers between different disease stages. Additional investigations evaluated the expression of CD162/PSGL-1 and CD106/VCAM-1, which play decisive roles in the tethering and adhesion of the monocytes to activated endothelium and their subsequent extravasation. While CD106/VCAM-1 expression increased only on classical CD14^++^CD16^−^ monocytes, CD162/PSGL-1 expression increased both on intermediate CD14^++^CD16^+^ as well as CD14^++^CD16^−^ monocytes. Previous studies have reported CD106 (VCAM-1) expression on tissue macrophages^22^,^23^. While monocyte subsets may differentially express the VCAM-1 receptor^24^, little data exists regarding the expression of VCAM-1 itself on circulating cells. Studies have reported its expression on various hematopoietic cells including cells of the myeloid lineage^25^.

MPO may contribute to atherogenesis^26^. Classical monocytes have high MPO expression^27^, and MPO-rich macrophages may contribute to destabilization and plaque rupture in acute coronary syndromes^26^. This study found that in addition to CD14^++^CD16^−^ classical monocytes, CD14^++^CD16^+^ intermediate monocytes also showed increased MPO expression in advanced stages of PAOD. MPO may thereby potentiate disease progression and may serve as an indicator of inflamed lesions. Of note, the similar rise of CD162/PSGL-1 and MPO in both classical and intermediate monocyte subsets supports Cros’s concept that both of these subsets share common functions^6^.

This study represents a single time point assessment, which poses a clear limitation. Determining monocyte subclasses during the natural history of disease as well as after therapy could provide further insight. Yet, of the 143 patients enrolled, 68 underwent angioplasty, and 44 underwent bypass surgery, leaving only 31 patients on non-interventional treatment, a cohort too small for substantial follow-up analyses. Similarly it has to be taken into account that the patient cohort reported included patients with coronary artery disease as well as carotid atherosclerosis, which may have influence on the monocyte subset distributions independently of the PAOD stages. We therefore compared monocyte subset distributions between patients with PAOD only and patients with generalized atherosclerosis (PAOD plus coronary and/or carotid artery disease). These results suggest that monocyte subsets rather indicate a general progression of atherosclerosis, as differences between the two cohorts were only moderate. This however needs to be further explored further study with larger patient cohorts. Methodologically, the analysis was performed on frozen samples. Freezing and thawing as well as the preparation of cells could significantly alter the cellular phenotype and potentially the subset distribution patterns. However, previous studies similarly worked with frozen monocyte samples, so the results are at least comparable^8^,^27^. Additional staining with a pan-monocyte marker such as CD45 or CD86 as recommended by Zawada *et al*. might have identified the monocyte population more clearly then on the FSC/SSC scatter alone^28^.

In summary, this study revealed substantial dynamics in monocyte subset distributions and phenotypes in different stages of PAOD. Intermediate CD14^++^CD16^+^ monocytes arose as the most promising biomarker for disease progression. This subset in addition showed a rise of the proatherogenic markers CD162/PSGL-1 and MPO with disease progression. MPO expression also increased in classical pro-inflammatory monocytes in advanced stages of PAOD. These observations not only expand insight into inflammatory aspects of PAOD, they identify monocyte subsets as novel potential therapeutic targets to decrease the inflammatory burden in advanced stages of peripheral atherosclerosis.

## Methods

### Patients

143 patients presenting to the vascular clinic for evaluation and treatment of femoropopliteal disease were prospectively and sequentially enrolled between 2012 and 2014. The study included patients with mild to severe PAOD, respectively suffering from intermittent claudication, rest pain to gangrene and tissue loss, representing Rutherford stages 1 to 6. Rutherford Stages were defined clinically as (I) mild claudication, (II) moderate claudication, (III) severe claudication, (IV) ischemic rest pain, (V) minor tissue loss and (VI) major tissue loss^29^. The study, which involved the biobanking of patient samples for further analysis, was approved by the Institutional review Board of the Technische Universität München, Munich, Germany (protocol number 5147/11). All patients gave informed consent before entering the study. All methods were in accordance with the relevant guidelines and regulations. The study was specifically carried out following the guidelines in the Declaration from Helsinki.

### Patient History and Examination

Thorough patient history was recorded upon enrolment. All patients received a clinical examination including treadmill testing, measurement of Ankle-brachial index (ABI), and duplex ultrasound (US), as well as a cross-sectional imaging study, using either Magnetic Resonance Angiography (MRA) or Computed Tomography Angiography (CTA) for therapy planning. Patient and lesion characteristics are shown in Table 1. Patients with active malignomas were not included in the study, similarly patients on dialysis were excluded.

### Sample Collection

Patient blood samples were obtained at the time of presentation and processed immediately to avoid coagulation and apoptosis. Venous blood samples were drawn in pyrogen-free tubes. To obtain leukocyte suspensions, whole blood was diluted 1:1 with DPBS and 20 ml diluted blood was overlaid on a 15 ml density gradient (Ficoll-Paque Plus, density 1.077 g/ml, GE Healthcare, NJ) and centrifuged (20 min, 1600 rpm, 18 °C). The mononuclear cell interphase was carefully isolated and washed 3 times with DPBS. Cell suspensions were frozen at −80 °C until further analysis.

Cell suspensions were stained with the following antibodies (all from BD Bioscience) at a final concentration of 1:100: CD11b-V450/ICRF44, CD14-PerCP-Cy5.5/M5E2, CD16 PE-Cy7/3G8, CCR2-Alexa-647/48607, CX3CR1-FITC/2A9-1, MPO-FITC/5B8, CD162-PE/KPL-1, and CD106-PE/51-10C9. Intracellular MPO staining was performed after fixation and permeabilization (BD Cytofix/Cytoperm, BD Bioscience, San Jose, CA). Cell phenotyping was performed using a Cyan Flow Cytometer (Beckman Coulter, Fichtenhain, Germany) after appropriate compensations. Flow cytometric data were analyzed using FlowJo v.8.5.2 (Tree Star, Inc., Ashland, USA).

### Statistics

Demographic patient data are presented as mean ± standard deviation, median and range, or as absolute and relative frequency. Comparisons of categorical demographic data between patients with different PAOD stages were performed using the Chi-square test or Fisher (-Freeman-Halton) exact test as appropriate. Continuous demographic data such as age, Body-Mass-Index (BMI), pain-free walking distance, and Ankle-Brachial-Index (ABI) were compared using Spearman’s correlation coefficient. Associations of the degree of atherosclerotic disease, as indicated by different Rutherford stages, with laboratory values were assessed using Pearson’s correlation coefficient. Additionally, to account for possible differences in relevant variables between patients in different Rutherford stages, partial correlation coefficients for the association between Rutherford stage and laboratory values of interest adjusted for age, sex, and risk factors (diabetes, tobacco abuse, hypertension and hyperlipidemia) were estimated. Data are presented in box plots. A p value < 0.05 was considered statistically significant. All statistical analyses were performed using Prism 6.0 (Graph Pad, La Jolla, USA).

## Additional Information

**How to cite this article**: Wildgruber, M. *et al*. The “Intermediate” CD14*++*CD16*+* monocyte subset increases in severe peripheral artery disease in humans. *Sci. Rep.* **6**, 39483; doi: 10.1038/srep39483 (2016).

**Publisher's note:** Springer Nature remains neutral with regard to jurisdictional claims in published maps and institutional affiliations.

## Supplementary Material

Supplementary Information

## Figures and Tables

**Table 1:** Patient characteristics.

Patient Characteristics	Rutherford 1 (n = 24)	Rutherford 2 (n = 8)	Rutherford 3 (n = 74)	Rutherford 4 (n = 16)	Rutherford 5 (n = 13)	Rutherford 6 (n = 8)	Total (n = 143)	p-value	r
Age (in years)*	73 (44–89)	71 (61–78)	70 (44–90)	65 (47–81)	74 (64–85)	82 (74–86)	72 (44–90)	0.190^§^	0.11
BMI (in kg/m^2^)*	27 (20–33.7)	26 (24–30)	26 (15–35.5)	24 (20–31)	26 (22–40)	20 (18–28)	26 (15–40)	0.106^§^	−0.162
Sex^**†**^								0.697^‡^	
Male	14 (58.3)	6 (75)	48 (64.9)	10 (62.5)	11 (84.6)	5 (62.5)	94 (65.7)		
Female	10 (41.7)	2 (25)	26 (35.1)	6 (37.5)	2 (15.4)	3 (37.5)	49 (34.3)		
**Risk factors**†									
Hypertension	20 (83.3)	8 (100)	65 (87.8)	12 (75)	13 (100)	7 (87.5)	125 (87.4)	0.309^‡^	
Tobacco use	5 (20.8)	4 (50)	24 (32.4)	8 (50)	0 (0)	2 (25)	43 (30.1)	**0.024**^‡^	
Diabetes	6 (25)	3 (37.5)	20 (27.03)	3 (18.75)	7 (53.85)	7 (87.5)	46 (32.2)	**0.006**^‡^	
Hyperlipidemia (LDL>160 mg/dl)	17 (70.8)	8 (100)	57 (77)	11 (68.8)	11 (84.6)	4 (50)	108 (75.5)	0.376^‡^	
Obesity	2 (8.3)	1 (12.5)	11 (14.9)	4 (25)	3 (23.1)	1 (12.5)	22 (15.4)	0.701^‡^	
**Other diseases**^**†**^									
Renal Insufficiency	3 (12.5)	3 (37.5)	13 (17.6)	5 (31.3)	5 (38.5)	2 (25)	31 (21.7)	0.211^‡^	
CAD	7 (29.2)	5 (62.5)	24 (32.5)	7 (43.8)	5 (38.5)	4 (50)	52 (36.4)	0.475^‡^	
Carotid disease	3 (12.5)	2 (25)	12 (16.2)	0 (0)	4 (30.8)	1 (12.5)	22 (15.4)	0.222^‡^	
Stroke	0 (0)	1 (12.5)	9 (12.2)	2 (12.5)	1 (7.7)	1 (12.5)	14 (9.8)	0.417^‡^	
Chronic heart failure	4 (16.7)	1 (12.5)	18 (24.5)	4 (25.1)	3 (23.1)	3 (37.5)	33 (23.1)	0.854^‡^	
Myocardial infarction	4 (16.7)	1 (12.5)	11 (15)	1 (6.3)	1 (7.7)	2 (25)	20 (14)	0.824^‡^	
Past history of Malignancy	4 (16.7)	5 (62.5)	14 (19)	1 (6.3)	0 (0)	3 (37.5)	27 (18.9)	**0.008**^‡^	
Aortic aneurysm	3 (12.5)	3 (37.5)	15 (19)	2 (12.5)	0 (0)	0 (0)	23 (16.1)	0.173^‡^	
**Medication**^**†**^									
Statins	6 (25)	7 (87.5)	52 (70.3)	9 (56.3)	8 (61.5)	5 (62.5)	87 (60.8)	**0.001**^‡^	
ACE inhibitors	8 (33.3)	2 (25)	26 (35.1)	5 (31.3)	4 (30.8)	5 (62.5)	50 (35)	0.751^‡^	
ß-blockers	6 (25)	3 (37.5)	28 (37.8)	8 (50)	4 (30.8)	5 (62.5)	54 (37.8)	0.507^‡^	
Cilostazol	2 (8.3)	0 (0)	7 (9.5)	0 (0)	3 (23.1)	0 (0)	12 (8.4)	0.380^‡^	
Coumadin	1 (4.2)	0 (0)	7 (9.5)	2 (12.5)	2 (15.4)	3 (37.5)	15 (10.5)	0.196^‡^	
Thrombocyte aggr. inhibitors	16 (56.7)	7 (87.5)	63 (85.1)	12 (75)	10 (76.9)	8 (100)	116 (81.1)	0.175^‡^	
**Bilateral disease**	6 (25)	1 (12.5)	10 (13.5)	2 (12.5)	1 (7.7)	0 (0)	20 (14)	0.633^‡^	
**Walking distance**	500 (300–1000)	285 (100–300)	100 (10–200)	35 (0–300)	x	x	100	**0.000**^§^	−0.767
Ankle brachial index*									
before excercise	0.76 (0.31–1.14)	0.68 (0.5–1)	0.63 (0.25–1.06)	0.58 (0.4–1.29)	0.73 (0.35–1.5)	0.56 (0.29–1.22)	0.67 (0.25–1.5)	**0.041**^§^	−0.176
after excercise	0.72 (0.4–1.07)	0.52 (0.29–0.74)	0.41 (0.19–0.93)	0.44 (0.38–0.71)	x	x	0.46 (0.19–1.07)	0.062^§^	−0.269

^*^Data as median and range.

^†^Data are numbers of patients; numbers in parentheses are percentages.

^‡^*P* value obtained with Fisher’s exact test.

^§^*P* value obtained with Pearson Correlation.

^II^P-value obtained with Chi-Square test.
