# Supplementary material for: The “Intermediate” CD14++CD16+ monocyte subset increases in severe peripheral artery disease in humans
Source: Sci Rep. 2016 Dec 19;6:39483. doi: 10.1038/srep39483 (PMC5171878; doi:10.1038/srep39483)
Supplement: Supplementary Information [file srep39483-s1.pdf]

Supplementary

The "Intermediate" $CD14^{++}CD16^{+}$ monocyte subset increases in severe peripheral artery disease in humans

Moritz Wildgruber, Teresa Aschenbrenner, Heiko Wendorff, Maria Czubba, Almut Glinzer, Bernhard Haller, Matthias Schiemann, Alexander Zimmermann, Hermann Berger, Hans-Henning Eckstein, Reinhard Meier, Walter A. Wohlgemuth, Peter Libby, Alma Zernecke

Supplementary Figure

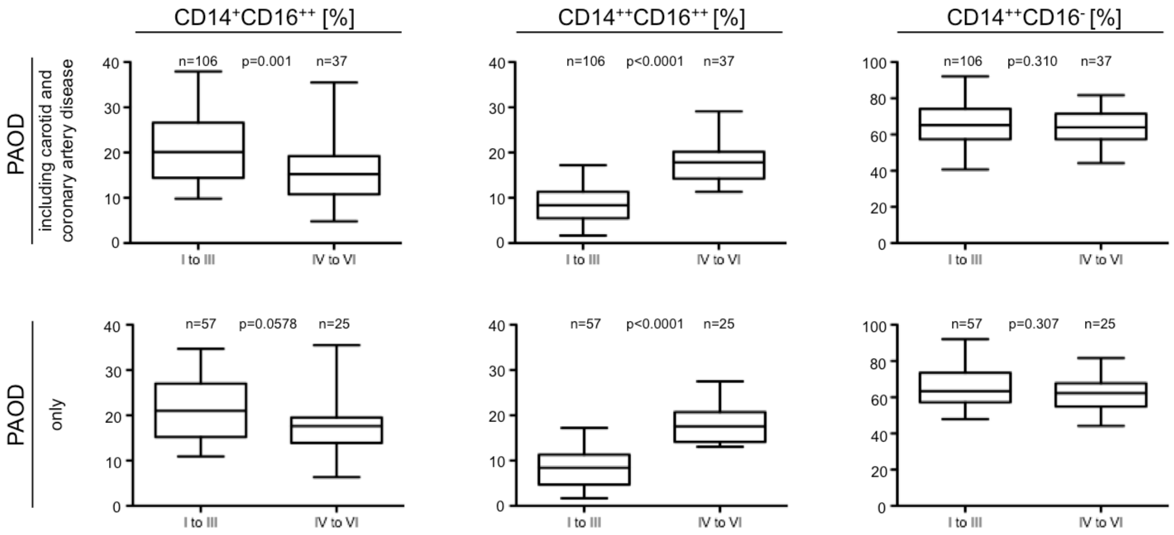

Supplementary Figure 1: ***Monocyte subset distributions between pooled disease stages (Rutherford stage I to III compared to stage IV to VI)***

Human monocyte subset distributions were compared between patients with moderate PAOD (Rutherford stage I to III) and advanced stages (Rutherford IV to VI). Results are shown for patients with generalized atherosclerosis (PAOD including patients with concomitant coronary and/or carotid artery disease, first row) and patients with PAOD only (excluding patients with concomitant coronary and/or carotid artery disease, second row). Data are presented as box plots. Sample sizes are: Rutherford stage I-III: n=106 and stage IV –VI: n=37 for patients with generalized atherosclerosis, and Rutherford stage I-III: n=57 and stage IV–VI: n=25 for patients with PAOD only. Statistical comparisons were performed using an unpaired two-sided student's t-test.
